# New Optical Tools to Study Neural Circuit Assembly in the Retina

**DOI:** 10.3389/fncir.2020.00044

**Published:** 2020-08-06

**Authors:** Aline Giselle Rangel Olguin, Pierre-Luc Rochon, Arjun Krishnaswamy

**Affiliations:** Department of Physiology, McGill University, Montreal, QC, Canada

**Keywords:** retina, neural circuits, synaptic specificity, circuit development, recognition molecules

## Abstract

During development, neurons navigate a tangled thicket of thousands of axons and dendrites to synapse with just a few specific targets. This phenomenon termed wiring specificity, is critical to the assembly of neural circuits and the way neurons manage this feat is only now becoming clear. Recent studies in the mouse retina are shedding new insight into this process. They show that specific wiring arises through a series of stages that include: directed axonal and dendritic growth, the formation of neuropil layers, positioning of such layers, and matching of co-laminar synaptic partners. Each stage appears to be directed by a distinct family of recognition molecules, suggesting that the combinatorial expression of such family members might act as a blueprint for retinal connectivity. By reviewing the evidence in support of each stage, and by considering their underlying molecular mechanisms, we attempt to synthesize these results into a wiring model which generates testable predictions for future studies. Finally, we conclude by highlighting new optical methods that could be used to address such predictions and gain further insight into this fundamental process.

## Introduction

Our mental abilities depend critically on the concerted function of many distinct neural circuits. While each is functionally specialized and therefore computationally separate from its neighbors, their wiring entangles them and embeds their synapses at micrometer scales. This proximity benefits function for a variety of reasons, ranging from a need to share neurons across circuits to a need to fit the brain’s wiring within the fixed volume of the skull. However, this dense arrangement poses serious challenges for circuit assembly because it requires developing neurons to select appropriate synaptic targets from several equally proximate alternatives. This selectivity creates specific wiring, but how a developing neuron chooses its synaptic partners is not entirely clear.

One possibility is that synaptic partners are genetically pre-programmed to synapse, an idea that came out of pioneering studies by Sperry (1943) and Langley (1892). Their vision was that axons possess ligands that match up with cognate receptors on targets and initiate the process of synaptogenesis. Their hypothesis, termed chemoaffinity, received enormous attention in the decades following its proposal and, in many cases, accurately predicts the synapses formed by developing neurons. *Sperry and Langley’s* chemical matchmakers would turn out to belong to large families of cell adhesion molecules, whose properties let neurons adhere tightly with appropriate targets (Sanes and Yamagata, 2009; Zipursky and Sanes, 2010; Lefebvre et al., 2015). Yet, concern grew over the completeness of this idea because the genome encodes too few of these “recognition” molecules to specify every unique connection in the nervous system. How is this disparity resolved?

One solution could be to use a single molecule to match partners separated by anatomical space or developmental time. In this scenario, each reuse subtracts from the total number of recognition molecules needed to wire a population of neurons. Another solution could be to use combinations of recognition molecules to match synaptic partners. Here, the power of combinatorics generates the required number of synaptic matchmakers from a handful of unique recognition molecules. Yet another solution could be to use recognition molecules to position partners in geometric arrangements that lead to specific matching. Recent work indicates that all these solutions are used in concert to simplify the demands on the genome and assemble neural circuitry.

Here, we review the evidence in support of these wiring rules, taken from recent work in the mouse retina. These studies outline a model in which specific wiring develops through a series of stages that include the directed axonal and dendritic growth, the formation of neuropil layers, the positioning of such layers, and the matching of co-laminar synaptic partners. Each section below focuses on one of these stages, the molecular mechanisms that guide each step and, when possible, parallels to layered circuitry in other brain regions and species. Next, we synthesize these recent findings into a series of open questions to help focus future inquiry, and, finally, we conclude by highlighting new optical methods that could be used to gain insight into this fundamental process.

## The Retina

**Figure 1:**
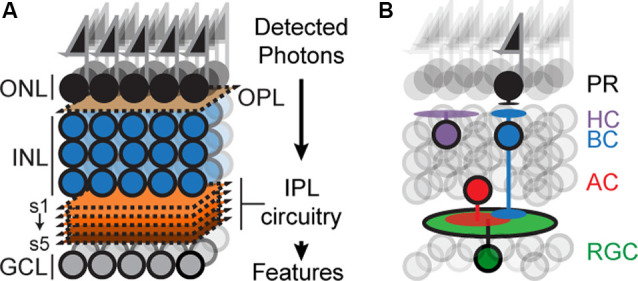
Schematicof retinal organization. **(A)** Outer and inner nuclear layers (ONL, INL), as well as ganglion cell layer (GCL), are separated by two neuropil layers called an outer plexiform layer (OPL) and an inner plexiform layer (IPL). The IPL can be subdivided into at least five sublayers or sublaminae (s1–s5). **(B)** Schematic showing the position of the principal cell types concerning overall retinal structure: photoreceptors (PR), bipolar (BCs), horizontal (HC), amacrine (AC), and retinal ganglion cells (RGC). Feature selectivity on RGCs arises from connections with a subset of BC and AC types.

The mouse retina has recently emerged as an attractive model for circuit development (Fuerst et al., 2009; Sanes and Zipursky, 2010; Matsuoka et al., 2011b; Wei et al., 2011; Lefebvre et al., 2012; Sun et al., 2013; Duan et al., 2014; Krishnaswamy et al., 2015). The retina is a thin sheet of neural tissue located at the back of the eye, which can be accessed with ease but is a part of the central nervous system (CNS) and contains many of the anatomical, cellular, and molecular features of circuits in the brain. The retina is composed of six principal cell types, arranged in three nuclear layers: Photoreceptors (PR) reside in the outer nuclear layer, interneurons called horizontal, amacrine and bipolar cells (HZs, ACs, and BCs) and Müller glia reside in the inner nuclear layer (INL), and retinal ganglion cells (RGCs) reside in the ganglion cell layer (GCL; Figure 1A). The outer, inner, and GCLs are separated by two specialized neuropils: an outer plexiform layer (OPL) containing synapses between PR, HZs and BCs, and an inner plexiform layer (IPL) containing synapses among RGCs, BCs and ACs (Masland, 2001, 2012; Sanes and Zipursky, 2010).

Decades of studies show that RGCs, ACs, and BCs can be divided into subtypes, each possessing characteristic anatomy, visual response, and molecular expression profile (Gollisch and Meister, 2010; Masland, 2012; Sanes and Masland, 2015). Many of the ~30 RGC, ~130 AC and ~13 BC types can be marked with type-specific antibodies and a full list of such markers is within sight due to an increasingly complete molecular taxonomy of the retina (Wässle et al., 2009; Macosko et al., 2015; Shekhar et al., 2016; Rheaume et al., 2018; Peng et al., 2019; Tran et al., 2019). Furthermore, many marker genes have been targeted by transgenic approaches to create an increasingly complete encyclopedia of cell-type-specific mouse lines housed at commercial repositories. Together, these discoveries make the retina among the few regions of the CNS where neural circuits can be perturbed, analyzed, and dissected with ease.

Retinal circuitry endows each RGC type with a unique preference for features in the visual scene such as edges, color, motion, and so on (Gollisch and Meister, 2010; Jadzinsky and Baccus, 2013; Sanes and Masland, 2015). The plan of such circuits is well understood (Figure 1B). Each circuit begins with a photoreceptor that detects photons and each ends with an RGC that sends visual information to the brain (Gollisch and Meister, 2010; Sanes and Zipursky, 2010; Masland, 2012; Sanes and Masland, 2015). Photoreceptor signals are relayed to the IPL *via* BCs which form glutamatergic synapses on RGCs and ACs; ACs form glycinergic or GABAergic synapses on RGCs, BCs, and/or other ACs depending on the cell type. Preferences for visual features arise because each RGC type receives input from a specific subset of BC and AC types, but how such subsets become wired is not completely understood.

## IPL Formation

Connections among RGCs, BCs, and ACs are organized in the IPL. The dendrites and axons of each RGC, BC, and AC type grow into just one or perhaps two of the ~5 IPL sublayers. Each of these classes is born sequentially: RGCs are born first (E8–E17), alongside ACs (E8–P5), followed by BCs (E17–P6), and Müeller glia (E14–P8; Young, 1985; Voinescu et al., 2009).

Birth, migration, and targeting occur sequentially for each retinal neuron, which means that synaptic partners appear asynchronously in target lamina. The direction-selective circuit in the retina offers a good illustration of this phenomenon: It is composed of connections among direction-selective ganglion cells (DSGCs), two populations of cholinergic starburst amacrine cells (SACs), and at least four subtypes of bipolar cells (Helmstaedter et al., 2013; Ding et al., 2016). Their interconnections are contained within IPL sublaminae 2 and 4, but each cell invades sublamina 2 and 4 at different times. SACs arrive by P1 (Stacy and Wong, 2003; Lefebvre et al., 2015; Ray et al., 2018), DSGCs by P5 (Kim et al., 2010; Ray et al., 2018), and BCs at P7–10 (Lefebvre et al., 2012; Duan et al., 2014). Specific wiring, therefore, does not depend on the synchronous appearance of these partners within the IPL. Instead, the sublaminae are used as meeting points; each neuron is endowed with information about the sublamina position, grows into these areas, and waits until its eventual synaptic partner arrives. Cell-type-specific ablation in zebrafish retinae further emphasize the widespread use of this strategy; loss of RGCs, ACs, Müeller glia, or combinations of these types delays the layer-specific growth of the spared neurons but does not prevent it (Kay et al., 2004; Randlett et al., 2013). It is an efficient strategy because it means that developmental mechanisms do not need to coordinate the birth, migration, and ingrowth of eventual partners to ensure their synapses. Rather, each cell is programmed with the laminar position of its eventual partner. How do these sublamina form?

### Repulsive Molecular Cues Help Define the IPL

The absence of somata in the IPL is among the most striking features of the retinal organization (Figure 1A). They are absent at the onset of IPL development, suggesting that somata and neurites are actively repelled from one another. Recent work shows that this separation is driven, in part, by members of the Semaphorin family.

Semaphorins (Semas) and their Plexin receptors (Plex) belong to a highly conserved protein family with important roles in dendritic and axon growth, including axon guidance, polarization, and repulsion (Huber et al., 2003). Sema-Plex interactions take on two forms: in one case, secreted Semas interact directly with Plex receptors on developing neurites, in the other, membrane-bound Semas interact with Plex receptors and its co-receptor, neuropilin. In both cases, activated Plex receptors cause growth cone collapse and repel Plex-expressing neurites from Sema expressing substrates (Huber et al., 2003). The retina expresses several Sema and Plex isoforms (Matsuoka et al., 2013; Zhang et al., 2017) and two pairs of these, Sema5A and 5B, and their receptors PlexA1 and A3, play an essential role in defining the IPL.

Sema5A and 5B mRNA is expressed broadly by neurons in the INL during retinal synaptogenesis (P3–P14) and disappears from these cells by P21. Their receptors, PlexA1 and A3, adopt a complementary expression pattern and broadly label axons and dendrites in the IPL. In the absence of Sema5A, Sema5B, PlexA1, or PlexA3, axons, and dendrites are unconfined to the IPL, often growing into the INL where they form ectopic IPL-like structures or growing through the INL to invade the outer nuclear layer (Matsuoka et al., 2011a). These ectopic projections are not the result of mistargeted IPL projections, rather these projections result from new processes that emanate from INL neurons. Thus, Sema-Plex signaling prevents these inappropriate processes from forming, thereby reinforcing a pre-existing attraction to the IPL. Little is known about the identity of such attractive cues, but the atypical cadherin FAT3 could be a potential candidate. Loss of FAT3 causes ACs to extend processes within the INL like they do in Sema/Plex mutants (Deans et al., 2011). The presence of such neurites might reflect an inability of Sema/Plex signaling to repel large numbers of mistargeted neurites or reflect an absence of Plex receptors on FAT3 ACs. Determining how attractive FAT3 signals coordinate with repulsive Sema/Plex signals requires the identity of FAT3-expressing neurons and the identity of the FAT3 ligand; both are currently unknown. Taken together, a balance of attractive and repulsive cues appears to separate dendrites and axons from somas to create the IPL.

Interestingly, the ectopic IPL structures formed in Sema/Plex mutants can recruit processes to form sublamina, even though they are located in the INL. These results suggest that sublaminar assembly and IPL formation are guided by independent molecular mechanisms.

### Attractive Cues Bind Growing Neurites Together to Create Sublamina

A recent study sheds new light on such sublaminar assembly mechanisms by analyzing the way SACs establish their layers in the developing mouse retina (Ray et al., 2018). One population of SACs resides in the INL and extends processes into sublamina 2. The other resides in the GCL and extends processes into sublamina 4. Each forms these sublayers at P0-P1, well before their BC and RGC synaptic partners arrive (Stacy and Wong, 2003; Sun et al., 2013; Ray et al., 2018).

By carefully analyzing the morphology of newly migrated INL SACs, Ray et al. (2018) observed that neighboring SACs contact each other, forming an initial plexus before they extend neurites into the IPL. Such contacts require a transmembrane protein called Megf10, whose loss ablates the inter-SAC contacts and, surprisingly, disrupts SAC lamination in sublamina 2 and 4; a similar disruption of SAC lamination was caused by ablating SACs earlier in development. Specifically, these manipulations cause SAC dendrites to diffuse out of their sublamina, disrupting the fine structure of these layers and creating ectopic SAC layers in adjacent sublamina (Ray et al., 2018). Exactly how the initial plexus ensures the proper formation of SAC laminae is unclear. However, its appearance coincides with a switch from a multipolar, migratory SAC morphology to one where SAC dendrites are oriented towards the IPL. Thus, inter-SAC contacts, sensed by Megf10, at this stage may act as a checkpoint that ensures SAC dendrite growth only when their cell bodies are positioned beside the IPL. Interestingly, the ectopic layers formed in Megf10 mutants can recruit the processes of SAC synaptic partners. For example, both direction-selective RGCs (DSGC) and BC projections colocalize with aberrant SAC projections in Megf10 nulls just as they would in controls, suggesting that partner recruitment is regulated independently of Megf10-dependent layer formation. The errors, however, are too severe to preserve DSGC function and result in a loss of their direction-selective responses. Thus, Megf10-based signals are critical for SACs to form an early pair of laminae, which serve as a substrate for their eventual synaptic partners.

Whether each retinal type possesses its own Megf10-like mechanism to establish sublamina or whether this mechanism is unique to SACs is not clear. In the case of the latter scenario, the pair of SAC laminae might act as reference points that later-born neurons could use to position their arbors. The observation that ectopic SAC laminae can recruit the processes of later-arriving neurons favors this possibility.

## Sublamina Selection

The sublaminar location of RGC or AC dendrites is a major determinant of their function. For example, RGCs become responsive to light onset (ON) because they synapse with BCs located in the inner half of the IPL; RGCs responding to light offset (OFF) synapse with BCs in the outer half (Figure 1B; Masland, 2012). ON or OFF BC/RGC pairs must, therefore, select a common sublamina in which to meet and synapse. How does such sublaminar selection occur? Recent work on Cadherin (Cdh) superfamily members in neurons of the retinal direction-selective circuit indicate that Cdhs play a crucial role in this process.

### Cdhs Target Neurons to Appropriate Layers

With a few exceptions, Cdhs are single-pass transmembrane proteins named for a characteristic calcium-dependent binding motif in their ectodomains and an intracellular domain that transmits binding events to the cell’s interior (Takeichi, 1988). Typically, a Cdh isoform on one cell will preferentially bind to the same isoform located on another cell. Such homophilic adhesion is an attractive property because it could be used to force Cdh-matched neurons to synapse specifically (Hatta et al., 1987; Suzuki et al., 1991; Inoue et al., 1998; Miskevich et al., 1998). Early studies lent support to this idea and showed that Cdh6^+^ or Cdh8^+^ cortical areas receive projections from Cdh6^+^ or Cdh8^+^ thalamic inputs, respectively (Suzuki et al., 1997). A similar matching was observed between Cdh9^+^ mossy fibers and their Cdh9^+^ CA3 targets in the hippocampus. Loss of Cdh9 in either mossy fibers or CA3 neurons selectively reduces their synaptic connectivity (Williams et al., 2011), suggesting that Cdh9 wires these populations together. Thus, Cdhs are expressed in complementary subsets of pre- and postsynaptic neurons and are important for these subsets to synapse with one another.

Recent work in the mouse retina extends this idea further and shows that two types of BCs, called BC2 and BC5, express specific Cdhs to find IPL sublamina containing their synaptic targets (Duan et al., 2014). BC2s express Cdh8 and grows into sublamina 2, BC5s express Cdh9 and grows into sublamina 4. Overexpression of Cdh8 in BC5 forces its growth into sublamina 2 instead of 4, the opposite was true when Cdh9 was expressed in BC2s, forcing these cells to grow into sublamina 4 instead of 2. Such misexpression experiments were able to redirect BC arbors regardless of whether the endogenous Cdh was present and could even redirect a Cdh-AC to the corresponding sublamina. Thus, Cdh8 and 9 impart layer position information for developing BCs. Interestingly, BC2s in Cdh8 nulls and BC5s in Cdh9 nulls distributed themselves randomly to both sublamina 2 and 4, suggesting that Cdh8 and 9 force BCs to choose between two equally attractive sublaminar locations.

Sublamina 2 and 4 contain the dendrites of DSGCs and SACs. DSGCs integrate OFF and ON BC excitation with SAC inhibition to detect the motion direction of an edge in the visual field; four ON-OFF DSGC subtypes exist, one for each cardinal direction (ventral, dorsal, temporal, nasal). Given this and given that BC2s are OFF-BCs and BC5s are ON-BCs, the authors next asked how Cdh loss impacts DSGC function.

To address this, the authors devised an optogenetic approach in which they recorded from fluorescently labeled DSGCs while delivering two-photon excitation to hundreds of individual Channelrhodopsin-2 (ChR2) expressing BC2s or BC5s (Duan et al., 2014; Krishnaswamy et al., 2015). In controls, ~50% of BC2s or BC5s whose axons overlapped DSGC dendritic arbors evoked inward glutamatergic currents. Loss of Cdh8 reduced this convergence from BC2s to fewer than 10%; loss of Cdh9 produced an equivalent loss of convergence from BC5s. Such deficits severely disrupt DSGC function—OFF or ON DSGC responses are effectively ablated by the loss of Cdh8 or 9, respectively. Oddly, these deficits occur even though Cdh-null BC2s and BC5s randomly distribute their axons to either sublamina 2 or 4, where the dendritic arbors of DSGCs reside. One reason for the increased severity of the functional deficit is that Cdh loss in these BCs prevents them from initiating synapse formation, possibly because they cannot adhere tightly to their DSGC targets. Testing this idea would require the identity of the Cdh8 and 9 ligands within sublamina 2 and 4. However, such ligands cannot be Cdh8 or Cdh9 themselves because these proteins are exclusively expressed by BC2 and BC5 respectively (Duan et al., 2014).

A follow-up study would identify a surprising set of culprits: a pair of isoforms closely related to Cdh9, called Cdh6 and 10, expressed selectively by SACs and a subpopulation of DSGCs selective for ventral motion (vDSGCs). All three of these Cdhs can bind heterophilically and, given their expression pattern, the authors asked whether their interactions bind the processes of BCs, DSGCs, and SACs together (Duan et al., 2018). A single and double knockout analysis produced no observable functional or anatomical deficit, which, together with expression studies, confirmed their suspicion that loss of one isoform could be compensated by another. Triple knockouts would be required but breeding such mice is impractical since Cdh6, 9, and 10 genes are arranged in tandem on chromosome 15. Using CRISPR-based genome editing to generate triple mutants (Basu et al., 2017; Duan et al., 2018), the authors discovered a surprising consequence of Cdh6/9/10 loss: the dendrites of vDSGCs diffused away from sublamina 2 and 4, but SACs dendrites were unaffected and laminated normally. Could SACs be using Cdhs to scaffold DSGC and BC projections instead of using them to grow into sublamina 2 and 4? To test this idea, the authors ablated SACs before IPL assembly and observed vDSGCs targeting deficits that phenocopied those seen in triple mutants (Duan et al., 2018). Without Cdh6, 9, and 10, vDSGCs are unable to find and synapse with SAC dendrites, rendering these vDSGC neurons unable to detect ventral motion.

Interestingly, sequencing studies revealed the expression of nine other Cdhs in cells of the direction-selective circuit, including Cdh7 in nasal motion selective DSGCs (nDSGCs) and its binding partner Cdh18 in SACs. Disrupting Cdh7 in nDSGCs with RNA interference led to synaptic targeting deficits that mimicked the phenotype seen in vDSGCs following Cdh6/9/10 deletion. Misexpression of Cdh18 in interneurons redirected arbors to Cdh7-positive DSGCs, indicating that Cdh7 binds to Cdh18 (Duan et al., 2018). Importantly, neither Cdh7 nor 18 perturbation nor the loss of Cdh6/9/10 alters SAC layering, further supporting the idea that SACs express Cdhs to scaffold projections from later-born neurons. Taken together, these results suggest that Cdh expression among DSGCs, BCs, and SACs directs these cells to a common pair of layers and creates direction-selective circuitry.

The scaffold strategy has the advantage of robustness; neurons that enter a layer using a Cdh can use the same Cdh to recruit later-laminating neurons. How the appropriate spatiotemporal combination of Cdh isoforms is regulated in targeting and scaffolding cells is unclear, but likely involves type-specific transcriptional regulation. Relating Cdh-expression patterns to transcription factor expression in single-cell sequencing atlases offer a way to uncover such factors.

### Semaphorins Repel Neurons From Inappropriate Layers

Adhesive mechanisms ensure contact between eventual partners, which recruits synapse formation machinery, and eventually leads to functional properties in circuits. However, what prevents inappropriate synapses that could disrupt function? For example, if OFF-RGCs received erroneous ON BC inputs, their responses to dark objects would be reduced or ablated. Controlling which neurons synapse after initial contact may be difficult because such sites are rapidly stabilized by transsynaptic interactions scaffolded by nascent pre- and postsynaptic organizers. Indeed, such powerful interactions can lead to remarkably normal-looking synapses even if they are between neurons with mismatched neurotransmitter and neurotransmitter receptor, or between a neuron and itself (Bekkers and Stevens, 1991; Sanes and Yamagata, 2009; Hassan and Hiesinger, 2015; Krishnaswamy, 2016). An alternate strategy is to avoid initial contact altogether by physically segregating inappropriately matched neurons.

Recent studies provide support for this view and show that segregation of ON and OFF neurons depends in part on repulsive Semaphorin signaling. Sema6a labels the dendrites of ON types, whereas its receptors, PlexA4 and PlexA2, are expressed on subsets of OFF types. Loss of Sema6A or PlexA4 causes AC and RGC processes that normally reside in OFF layers to reside in the ON layers instead. For example, loss of PlexA4 leads sublamina 1-preferring melanopsin positive RGCs and tyrosine-hydroxylase positive ACs to grow into sublamina 5 (Matsuoka et al., 2011a). A similar abnormality is seen in the absence of Sema6a, suggesting that Sema6a-PlexA4 binding restricts melanopsin positive RGC and tyrosine-hydroxylase positive AC projections to sublamina 1. Interestingly, these abnormal, sublamina 5-located RGC and AC projections still colocalize with one another, suggesting that these neurons still synapse despite their ectopic location. In another example, OFF SACs in PlexA2 mutants form aberrant projections to sublayers normally reserved for ON SACs; this mistargeting impairs direction selectivity because OFF motion signals are inappropriately combined with ON motion signals (Sun et al., 2013).

## Target Selection

Laminae simplify wiring complexity because they place eventual partners close by and inappropriate ones far apart (Sanes and Yamagata, 2009; Baier, 2013). A potential drawback of this arrangement is that late-born neurons must find their targets within layers that are increasingly crowded. How do they do this? An initial hypothesis proposed that all neurons within a lamina connect, with the number of type-specific synapses scaling in proportion to contact frequency. However, several connectomic efforts indicate that there is no correlation between how widely two neurons contact each other and how often they synapse (Briggman et al., 2011; Helmstaedter et al., 2013; Kasthuri et al., 2015). Instead, such studies indicate that neurons synapse on target cells whose membranes are within microns of the membranes of several non-target cells. How does such selectivity arise?

### Immunoglobulin Superfamily Members Enrich Connections Among Synaptic Partners

An increasingly likely scenario is that eventual partners can recognize each other at a micrometer scale because they express matching recognition molecules from the immunoglobulin superfamily (Ig). There are ~500 Igs in mice, whose names refer to their extracellular domains, which have similarities to the antigen-combining site of antibodies (Shapiro et al., 2007; Katidou et al., 2008; Baier, 2013). Igs typically interact homophilically, but heterophilic interactions among some isoforms have been described. They are expressed in complex combinatorial patterns in the retinae of several species and are of interest because of their recently uncovered roles in wiring co-laminar neurons.

In the fly retina, a multilayered IPL-analog called the medulla organizes indirect input from PR across several sublaminae to create circuits selective for features such as motion direction (Sanes and Zipursky, 2010). Indirect input cells, called laminar neurons grow into specific medullary layers and synapse with specific interneurons or projection neuron types. These type-specific connections are not exclusive; laminar neurons form large numbers of synapses with their targets, but synapse at low levels with nearby cells. Thus, specificity reflects enriched connectivity between laminar and medullary cell types. An Ig family of defective probosci’s proteins (Dprs) and their receptors from the Dip family are essential for this enrichment (Tan et al., 2015; Xu et al., 2018). Laminar neurons expressing a Dpr isoform form numerous synapses with medullary targets that express cognate Dips. Loss of a Dpr reduces selective synapses between the laminar neuron and its Dip-matched medullary partner with minimal effect on layer selection or gross morphology (Xu et al., 2019). Consistent with this, Dpr misexpression in neurons that overlap a Dip-matched target promotes synapses between the pair but cannot promote synapses with non-overlapping neurons (Courgeon and Desplan, 2019; Menon et al., 2019; Xu et al., 2019). Thus, Ig interactions in flies bias connectivity between physically proximate neurons in favor of appropriate pairings.

New work indicates a similar role for Igs in vertebrates. A recent study shows that the Ig member Sidekick 2 (Sdk2) enriches connections among co-laminar ACs and RGCs in the mouse retina. The two Sdks in mice were named for the related Sdk gene in Drosophila (Nguyen et al., 1997; Astigarraga et al., 2018). They are large (~250 kD) single-pass transmembrane proteins with six immunoglobulin domains, thirteen fibronectin repeats, and a cytoplasmic domain containing a PDZ binding motif. The PDZ anchors Sdks to nascent synapses where they have been shown to interact with the Magi family of scaffold proteins (Yamagata and Sanes, 2010). Sdks bind homophilically across cell-cell junctions *via* their Ig domains (Yamagata and Sanes, 2010; Krishnaswamy et al., 2015), raising the possibility that Sdk expression in co-laminar AC and RGC subsets could enrich their connectivity.

To test this idea, Krishnaswamy et al. (2015) probed Sdk expression in the mouse retina and found that Sdk2 labels an RGC called W3B and an AC called VG3. Both neurons overlap extensively in the same sublamina, each forming small-diameter, highly branched dendritic arbors. W3B-RGCs are thought to be object motion detectors and fire action potentials if motion in its narrow receptive field center differs from a motion in its millimeter-sized surround. VG3-ACs are named for their expression of the non-classical vesicular glutamate transporter, VGlut3, and were a previously uncharacterized AC type (Grimes et al., 2011). The anatomical overlap between these two neurons and their shared expression of Sdk2 led Krishnaswamy et al. (2015) to ask whether VG3 and W3B synapse selectively with one another.

Using optogenetic mapping, the authors recorded from individual W3B-RGCs while stimulating hundreds of presynaptic VG3-ACs located at various distances from the patched cell. Stimulation of VG3-ACs evokes excitatory synaptic currents on W3B-RGCs that result from direct glutamatergic synapses. Comparing evoked current amplitude vs. inter somatic spacing for each VG3-W3B pair revealed an inverse relationship–the strongest responses were produced by the closest VG3-ACs, with the strength of these responses decreasing to no different than baseline at spacings greater than 100 μm. This threshold distance corresponds to the maximal spacing a VG3-AC and W3B-RGC can be positioned and still be in contact. On the other hand, every VG3-AC located within 100 μm of a W3B-RGC was functionally connected. This enriched connectivity pattern is specific to W3B-RGCs and VG3-ACs, although they have substantially weaker, spatially disordered connections with nearby cells. Importantly, none of these equally proximate alternatives express Sdk2, which led the authors to study the consequences of Sdk2 loss. Repeating the mapping experiment in Sdk2 knockout mice revealed pronounced deficits in connectivity–fewer than 10% of VG3-ACs synapse with W3B-RGCs in the absence of Sdk2, and what few were connected have amplitudes reduced to ~20% of that found in controls. This connectivity loss was restricted to VG3-ACs and W3B-RGCs and arose without major changes in the layering of these two cells. Rescuing Sdk2 expression in VG3 could not rescue VG3-W3B connectivity, indicating that Sdk2 homophilic interactions enrich VG3-W3B connectivity. Moreover, loss of VG3-AC connections in Sdk2 knockouts caused severe loss of excitatory drive to W3B-RGCs, impairing their ability to sense object motion. Taken together, these results indicate that Sdk2 biases connections among proximate partners. Intriguingly, a close relative of Sdk2, Sidekick 1, labels an interneuron-RGC pair that overlaps with W3B and VG3, suggesting that Sdk isoform choice may wire two functionally distinct circuits despite physical entanglement (Krishnaswamy et al., 2015; Yamagata and Sanes, 2018).

Earlier work in chicks suggested that Igs like Sdks direct laminar targeting, rather than synaptic bias (Yamagata et al., 2002; Krishnaswamy et al., 2015). Species-specific differences could explain this discrepancy and have been observed for close relatives of Sdks. For example, Down syndrome cell adhesion molecules (DSCAMs) direct laminar targeting in chicks (Yamagata and Sanes, 2008) but serve dendritic self-avoidance and cell survival functions in mice (Fuerst et al., 2008, 2009, 2012). Another possibility reflects experimental differences; in chicks, Sdk2 expression was reduced in isolated neurons in control retinae, which may have amplified their morphological deficits due to competition between the perturbed and wildtype neurons (Yamagata and Sanes, 2008). A final possibility suggested by a recent study in mouse retinae is that Igs can alter laminar morphology depending on when they are expressed. For example, early Sdk2 misexpression in Sdk1ACs causes their morphology to resemble VG3 and W3B (Yamagata and Sanes, 2018). Whether this is because Sdks regulate dendritic morphology, as has been shown for related Contactins (Peng et al., 2017), or because morphological changes result from increased Sdk1AC-W3B and/or Sdk1AC-VG3 synapses is not clear. More work will be needed to decide whether Igs bias connectivity because of when they are expressed, because of their intrinsic properties, or both. Whatever the mechanism, a growing number of studies strongly point to Igs as conserved neural wiring genes that direct local synapse formation.

## An Adhesive Code?

**Figure 2:**
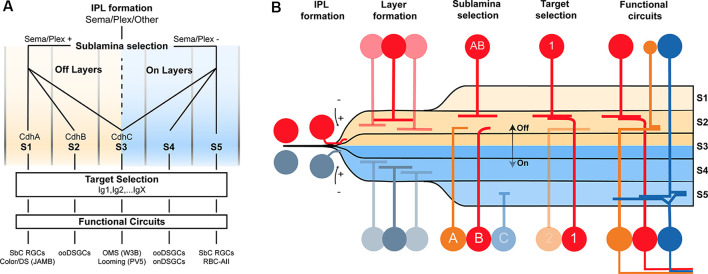
Potential model of the circuit assembly. A wiring model (A) and developmental stages (B) followed by developing retinal neurons to form functional neural circuits. **(A)** Speculative decision tree model that summarizes the studies reviewed in this text. The diagram has been populated with recognition molecule candidates with known roles at each stage. **(B)** Cartoon of circuit assembly in the mouse retina illustrating the stages of migration, IPL formation, layer formation, sublamina selection, and target selection which ultimately result in mature neural circuitry.

Much more work is needed to understand the basis of wiring specificity, but the following tentative conclusions would fit the evidence reviewed so far. First, specificity comprises at least three steps: layer formation, layer targeting, and intralaminar targeting. Second, neurons find appropriate layers even if their eventual targets have not arrived. Third, layer formation is orchestrated by diverse adhesive and repulsive molecular cues. Fourth, layer targeting is dominated by members of the Cdh and Sema/Plex families. Fifth, target selection is dominated by members of the Ig family (Figures 2A,B).

These conclusions outline a model in which the expression of a given Sema, Cdh, and Ig defines a neuron’s wiring pattern in the same way an address directs an envelope to a specific state/province, city, and mailbox (Figures 2A,B).

This model drastically reduces the number of wiring cues. For example, a Cdh that directs retinal neurons to an ON sublamina could be reused for an OFF sublamina, so long as the OFF-projecting cells are repelled from ON layers using Sema/Plex members. As another example, a single Sdk isoform could be used to connect several pairs so long as each pair expresses a different Cdh/Sema combination to target different lamina. The hierarchical nature of circuit assembly in the retina, paired with the diversity-generating process of combinatorics dramatically simplifies the genetic needs for wiring.

## Genetically Encoded Optical Tools to Gain Insight

### Anatomical Connectivity Mapping

Connections from subsets of interneuron types on a single RGC type create circuits, each attuned to a unique aspect of the visual scene. Current models predict that wiring patterns specific to each circuit arise because the component neurons make wiring decisions guided by recognition molecules. A satisfying understanding of this process requires that we test the ability of this model to predict connectivity patterns across all retinal circuits. However, such mature connectivity patterns for most retinal circuits are unknown. Straightforward, high-throughput methods to map connectivity are needed to cross this hurdle.

**Figure 3:**
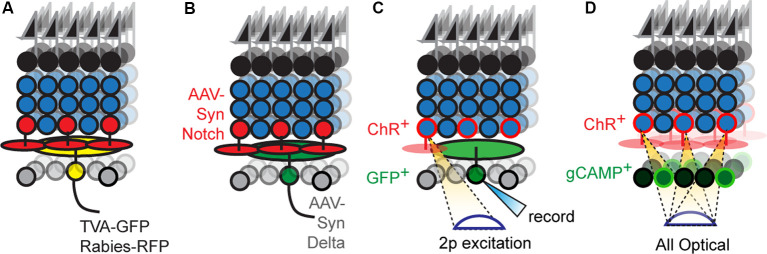
New optical tools to map retinal connectivity. **(A)** Retrograde infection with the rabies virus and cre-dependent TVA/rG allows tracing of monosynaptic connections from defined starter cells. **(B)** Putative synthetic delta constructs delivered retrogradely to RGCs alongside retinal infection with synthetic notch reveals connected interneuron types. **(C)** Two-photon excitation of ChR2^+^ interneurons while recording individual RGCs allows maps of convergence. **(D)** All-optical physiological methods permit divergence maps from defined interneuron types to be measured.

In principle, labeling a single RGC and its presynaptic partners followed by immunostaining with cell-type-specific markers offers a route to progress. Rabies-based transsynaptic tracing offers a way to achieve such labeling (Figure 3A). Initial attempts with this approach were hard to interpret because the virus would “hop” across synapses so rapidly that it was difficult to distinguish mono- vs. polysynaptically connected cells (Wickersham and Feinberg, 2012). The solution was to employ two viruses, mutated rabies incapable of transsynaptic infection that encodes a reporter, and an adeno-associated virus bearing cre-dependent rabies glycoprotein (rG), which permits transsynaptic transfer (Wickersham et al., 2007a). Injecting these two viruses into the medial terminal nucleus of mice retrogradely infects ON-DSGCs (Dhande et al., 2013) and ON-OFF DSGCs (Yonehara et al., 2011) and labels their monosynaptically connected interneuron inputs, such as SACs. A further refinement uses the two viruses above, but pseudotypes the rabies virus with avian capsid proteins, which restricts infection to neurons that bear avian rabies receptors, called TVA receptors (Wickersham et al., 2007b). Infecting medial terminal nucleus with viruses bearing TVA and rG, followed by injection of g-deleted rabies into the eye allowed for even more precise labeling of ON-DSGC inputs, revealing their connections with Type 5 BCs (Yonehara et al., 2013). Current viral reagents encode cre-dependent TVA and rG within a single virus which is used along with g-deleted rabies viruses. However, this approach still labels several cre-expressing starter neurons which in turn labels so many presynaptic neurons that it can overwhelm measurements of convergence on individual starters. This is a particularly troublesome feature for the retina since each circuit repeats itself across the retinal surface with a substantial lateral overlap in the wiring. Delivering TVA/rG to a single starter circumvents this issue at the cost of a loss in throughput (Schubert et al., 2018).

Another widely used strategy involves the cell-type-specific expression of wheat germ agglutinin (WGA) fused to GFP or Cre-recombinase. However, transfer with these genetically encoded tracers is not limited to monosynaptic inputs which dilute the WGA signal in directly connected neurons as the WGA diffuses through the network. Moreover, the transfer can be non-synaptic and can be biased to some synapses over others, resulting in erroneous wiring diagrams (Wickersham and Feinberg, 2012).

Newer strategies employ proteins that bind transsynaptically to label the synapses of connected neurons (Figure 3B). A good example is a GRASP, which reconstitutes GFP across synaptic partners (GRASP). Briefly, a membrane-bound, incomplete piece of GFP is expressed presynaptically while the missing piece of GFP is fused to a postsynaptically localized membrane protein (Feinberg et al., 2008). If the two neurons synapse, their membranes are close enough to unite the pieces and GFP fluorescently labels the synapse. This approach is widely used in invertebrates to label synapses, in part, because the well-studied neuronal morphologies in these systems allow one to translate the punctate signal into type-specific wiring diagrams. A few versions have been optimized for mice (Kim et al., 2011; Luo et al., 2018) and used in the retina (Yamagata and Sanes, 2012), but the wiring complexity in these systems makes it hard to assign synaptic labeling to specific pre- and postsynaptic neurons. For this reason, soma-filling labels that result from transsynaptic interactions have been devised, but at present are only available in invertebrate models (Jagadish et al., 2014; Talay et al., 2017). A recent study has used synthetic reporters based on the Notch-Delta signaling pathway (synNotch) in vertebrates (Morsut et al., 2016); in this pathway, Notch intracellular domain is cleaved following Notch-Delta binding and activates gene transcription. Synthetic versions replace Notch intracellular domains with Cre while replacing Notch and Delta ectodomains with GFP and GFP-nanobodies, respectively. The proximity produced by synaptic contact results in nanobody-GFP binding, which frees the cre-containing intracellular domain to activate reporter gene expression. Potential is the concern that normally unconnected neurons expressing either GRASP or artificial Notch reporters might erroneously synapse because these tools might act as adhesion proteins. Current reports using GRASP have carefully controlled for this possibility for the circuit under study, and at least in these cases, the likelihood of such artifactual synapses is low. Less is known about the behavior of Notch-based reporters which have used in culture (Morsut et al., 2016). A more comprehensive strategy to validate these methods could be to compare resulting wiring diagrams with those obtained using electron microscopy (EM) based connectomic methods. Such correspondence would be powerful, and with the advent of new EM compatible genetically encoded reporters, the labor cost of this approach is significantly reduced (Joesch et al., 2016).

Viral and genetically encoded tracers offer powerful avenues for labeling connected neurons. Their major drawback is sensitivity–presynaptic neurons that form a single synapse with a target are labeled just as brightly as those synapsing many times with the same target. This is a worrisome limitation given that specificity mechanisms may simply enrich connections between appropriate partners rather than preventing connections among inappropriate ones. Thus, an approach that measures connectivity strength would be ideal. One way to add this information to tracer-labeled circuits could be to process them for array tomography, which combines the ultra-thin sections employed in EM with the histological approaches common to light microscopic assays. Such EM-prepared sections can be stained with multiple rounds of antibodies that label synaptic proteins which results in an “array” of staining that identifies synapses between traced neurons. This approach has limitations–significant time is needed to process, image, and reconstruct tissue, which places practical limits on the number of samples one can acquire. An alternate track is to relate anatomically traced neurons to the strength of their functional connections.

### Functional Connectivity Mapping

A straightforward way to link physical and functional connectivity is to record synaptic transmission in one neuron while stimulating another and label each cell with different intracellular dyes (Figure 3C). Incorporating type-specific cre lines into this scheme permits connectivity analysis of specific interneuron-RGC pairs that include their spatial relationship.

This approach has proved invaluable to the study of retinal direction-selective circuits. Briefly, there are four types of DSGCs; each responds to stimuli moving in one of four cardinal directions (ventral, dorsal, nasal, and temporal). Their preference is established by SACs who inhibit DSGC responses to stimuli moving opposite the preferred direction, called the null direction; for example, dorsal motion responses are inhibited on DSGCs selective for ventral motion. Paired recording methods were essential to learning that these inputs were strongest on the null side of DSGC dendrites and that such strength develops over the first postnatal week (Wei et al., 2011). Optogenetic tools accelerate and simplify these studies, allowing activation of interneurons expressing channelrhodopsins (ChRs) in arbitrary patterns while recording from individual RGC types, allowing one to rapidly assess the strength and geometry of interneuron-RGC connections. The relative ease of this experiment, for example, allowed Yonehara et al. (2011) to rapidly measure the asymmetric GABAergic SAC input and symmetric cholinergic SAC input on dozens of individual DSGCs (Yonehara et al., 2011).

As a further refinement of this optogenetic strategy, Krishnaswamy et al. (2015) excited ChR^+^ interneurons with automated two-photon excitation, resolving interneuron-RGC connectivity maps at the level of individual pairs (Duan et al., 2014, 2018; Krishnaswamy et al., 2015). Across three studies, these authors comprehensively mapped the convergence between seven interneurons and four RGCs types and found several unique connectivity patterns, which could then be targeted by developmental approaches. For example, mapping BC2-DSGC and BC5-DSGC connectivity revealed that only about 50% of the BCs situated above the DSGC dendritic arbor were connected (Duan et al., 2014, 2018). Why this occurs is not clear but could be related to the array-like organization of BC axon terminals and the sparseness of DSGC dendritic arbors. Stimulating the terminals of ChR2^+^ BCs rather than their somas, using the same approach, could allow a direct test of this idea. In another study, two-photon mapping interneurons and RGCs that co-stratify in sublamina 3 revealed that only a few of these neurons exhibit strong connections; VG3-ACs and W3B RGCs connect whenever their dendrites overlap, whereas connectivity is absent between Nex-Cre labeled ACs and W3B-RGCs despite comparable anatomical overlap (Krishnaswamy et al., 2015).

One drawback of this approach is speed; two-photon mapping measures the strength and geometry of an interneuron’s connections to a single RGC with exquisite detail, but analysis of this interneuron’s connections to other nearby RGCs requires a separate experiment. This separation can be costly because of the significant time investment needed to generate mice that label a single AC type with ChR2 and single RGC type with GFP. This is an important consideration in light of data showing that Cdhs coordinate the growth of many RGCs to a common layer and bring them within proximity of several common interneuron partners. Determining whether this proximity causes such RGCs to share interneuron inputs would ideally require an approach that maps several interneuron-RGC pairs in parallel.

All-optical connectivity mapping approaches are a possible route for parallelization; in this scheme, ChR2^+^ interneurons are individually excited while simultaneously imaging the responses of all RGCs (Figure 3D). Briefly, a spatial light modulator is used to sculpt two-photon laser emission into holograms that are aimed at an arbitrary number of interneuron somas, while simultaneous two-photon scanning images responses from RGCs that express calcium indicators. The <2 μm axial resolution of this approach ensures that there is no crosstalk between stimulating and recording planes. Such all-optical methods have already been applied to cortical circuits with great success (Packer et al., 2015; Marshel et al., 2019).

Given the graded, non-spiking membrane properties of most interneurons, the application of this method to retinal circuitry is likely to be successful. Indeed, a preprint describing this approach in retina indicates that one can stimulate hundreds of rod BCs while measuring responses in a field of RGCs expressing genetically encoded calcium indicators (Spampinato et al., 2019).

This functional mapping offers a powerful way to define connectivity strength among synaptically coupled neurons. But combining these methods with the viral or genetic tracers described above may allow for even faster, higher-detail connectivity diagrams. For example, labeling the interneurons connected to an RGC with rabies viruses bearing ChRs could allow one to optogenetically map their connectivity onto the starter RGC as well as nearby non-starter RGCs. Comparisons across such maps could reveal systematically stronger connectivity strengths to the starter RGC relative to non-starter RGCs, directly testing the notion that specificity enriches synapses between partners rather than ensuring exclusive synapses between partners. A further improvement to this scheme would follow functional mapping with array tomographic or EM-based connectomic approaches to relate pair connection strength to synapse number, or synaptic molecular profile. A combination of functional, anatomical, and ultrastructural approaches is needed for a complete picture.

## Conclusion

A major goal of developmental neuroscience is to unearth the rules and blueprints used to wire the brain. Recent efforts to understand how the well-defined circuits of the mouse retina assemble are shedding new light on this issue. These studies outline a wiring model in which connectivity develops through a series of stages; a neuron’s route through these stages is governed by the expression of specific recognition molecules, which direct them to grow in a specific direction, to a specific layer, and synapse with a specific target. New advances in neurophotonics offer a way to accelerate our understanding of this process and develop a comprehensive model that relates recognition molecule expression to wiring patterns. By gaining these valuable insights in the retina and improving our circuit tracing toolkit, we ready ourselves to translate these advances to understand circuit assembly in the brain where genetically programmed wiring patterns are modified by neural activity.

## Author Contributions

AR, P-LR, and AK planned and wrote the manuscript.

## Conflict of Interest

The authors declare that the research was conducted in the absence of any commercial or financial relationships that could be construed as a potential conflict of interest.

## References

[B1] Astigarraga S. Douthit J. Tarnogorska D. Creamer M. S. Mano O. Clark D. A. (2018). *Drosophila* sidekick is required in developing photoreceptors to enable visual motion detection. Development 145:dev158246. 10.1242/dev.158246 29361567 PMC5818003

[B2] Baier H. (2013). Synaptic laminae in the visual system: molecular mechanisms forming layers of perception. Annu. Rev. Cell Dev. Biol. 29, 385–416. 10.1146/annurev-cellbio-101011-155748 24099086

[B3] Basu R. Duan X. Taylor M. R. Martin E. A. Muralidhar S. Wang Y. (2017). Heterophilic type II cadherins are required for high-magnitude synaptic potentiation in the hippocampus. Neuron 96, 160.e8–176.e8. 10.1016/j.neuron.2017.09.009 28957665 PMC5634529

[B4] Bekkers J. M. Stevens C. F. (1991). Excitatory and inhibitory autaptic currents in isolated hippocampal neurons maintained in cell culture. Proc. Natl. Acad. Sci. U S A. 88, 7834–7838. 10.1073/pnas.88.17.7834 1679238 PMC52398

[B5] Briggman K. L. Helmstaedter M. Denk W. (2011). Wiring specificity in the direction-selectivity circuit of the retina. Nature 471, 183–188. 10.1038/nature09818 21390125

[B6] Courgeon M. Desplan C. (2019). Coordination between stochastic and deterministic specification in the *Drosophila* visual system. Science 366. 10.1126/science.aay6727 31582524 PMC6819959

[B7] Deans M. R. Krol A. Abraira V. E. Copley C. O. Tucker A. F. Goodrich L. V. (2011). Control of neuronal morphology by the atypical cadherin Fat3. Neuron 71, 820–832. 10.1016/j.neuron.2011.06.026 21903076 PMC3521586

[B8] Dhande O. S. Estevez M. E. Quattrochi L. E. El-Danaf R. N. Nguyen P. L. Berson D. M. (2013). Genetic dissection of retinal inputs to brainstem nuclei controlling image stabilization. J. Neurosci. 33, 17797–17813. 10.1523/jneurosci.2778-13.2013 24198370 PMC3818553

[B9] Ding H. Smith R. G. Poleg-Polsky A. Diamond J. S. Briggman K. L. (2016). Species-specific wiring for direction selectivity in the mammalian retina. Nature 535, 105–110. 10.1038/nature18609 27350241 PMC4959608

[B10] Duan X. Krishnaswamy A. De la Huerta I. Sanes J. R. (2014). Type II cadherins guide assembly of a direction-selective retinal circuit. Cell 158, 793–807. 10.1016/j.cell.2014.06.047 25126785

[B11] Duan X. Krishnaswamy A. Laboulaye M. A. Liu J. Peng Y.-R. Yamagata M. (2018). Cadherin combinations recruit dendrites of distinct retinal neurons to a shared interneuronal scaffold. Neuron 99, 1145.e6–1154.e6. 10.1016/j.neuron.2018.08.019 30197236 PMC6284407

[B12] Feinberg E. H. Vanhoven M. K. Bendesky A. Wang G. Fetter R. D. Shen K. (2008). GFP reconstitution across synaptic partners (GRASP) defines cell contacts and synapses in living nervous systems. Neuron 57, 353–363. 10.1016/j.neuron.2007.11.030 18255029

[B13] Fuerst P. G. Bruce F. Rounds R. P. Erskine L. Burgess R. W. (2012). Cell autonomy of DSCAM function in retinal development. Dev. Biol. 361, 326–337. 10.1016/j.ydbio.2011.10.028 22063212 PMC3246579

[B14] Fuerst P. G. Bruce F. Tian M. Wei W. Elstrott J. Feller M. B. (2009). DSCAM and DSCAML1 function in self-avoidance in multiple cell types in the developing mouse retina. Neuron 64, 484–497. 10.1016/j.neuron.2009.09.027 19945391 PMC2850049

[B15] Fuerst P. G. Koizumi A. Masland R. H. Burgess R. W. (2008). Neurite arborization and mosaic spacing in the mouse retina require DSCAM. Nature 451, 470–474. 10.1038/nature06514 18216855 PMC2259282

[B16] Gollisch T. Meister M. (2010). Eye smarter than scientists believed: neural computations in circuits of the retina. Neuron 65, 150–164. 10.1016/j.neuron.2009.12.009 20152123 PMC3717333

[B17] Grimes W. N. Seal R. P. Oesch N. Edwards R. H. Diamond J. S. (2011). Genetic targeting and physiological features of VGLUT3+ amacrine cells. Vis. Neurosci. 28, 381–392. 10.1017/s0952523811000290 21864449 PMC4150031

[B18] Hassan B. A. Hiesinger P. R. (2015). Beyond molecular codes: simple rules to wire complex brains. Cell 163, 285–291. 10.1016/j.cell.2015.09.031 26451480 PMC4600127

[B19] Hatta K. Takagi S. Fujisawa H. Takeichi M. (1987). Spatial and temporal expression pattern of N-cadherin cell adhesion molecules correlated with morphogenetic processes of chicken embryos. Dev. Biol. 120, 215–227. 10.1016/0012-1606(87)90119-9 3817290

[B20] Helmstaedter M. Briggman K. L. Turaga S. C. Jain V. Seung H. S. Denk W. (2013). Connectomic reconstruction of the inner plexiform layer in the mouse retina. Nature 500, 168–174. 10.1038/nature12346 23925239

[B21] Huber A. B. Kolodkin A. L. Ginty D. D. Cloutier J.-F. (2003). Signaling at the growth cone: ligand-receptor complexes and the control of axon growth and guidance. Annu. Rev. Neurosci. 26, 509–563. 10.1146/annurev.neuro.26.010302.081139 12677003

[B22] Inoue T. Tanaka T. Suzuki S. C. Takeichi M. (1998). Cadherin-6 in the developing mouse brain: expression along restricted connection systems and synaptic localization suggest a potential role in neuronal circuitry. Dev. Dyn. 211, 338–351. 10.1002/(SICI)1097-0177(199804)211:4<338::AID-AJA5>3.0.CO;2-I 9566953

[B23] Jadzinsky P. D. Baccus S. A. (2013). Transformation of visual signals by inhibitory interneurons in retinal circuits. Annu. Rev. Neurosci. 36, 403–428. 10.1146/annurev-neuro-062012-170315 23724996

[B24] Jagadish S. Barnea G. Clandinin T. R. Axel R. (2014). Identifying functional connections of the inner photoreceptors in drosophila using tango-trace. Neuron 83, 630–644. 10.1016/j.neuron.2014.06.025 25043419 PMC4126867

[B25] Joesch M. Mankus D. Yamagata M. Shahbazi A. Schalek R. Suissa-Peleg A. (2016). Reconstruction of genetically identified neurons imaged by serial-section electron microscopy. Elife 5:e15015. 10.7554/eLife.15015 27383271 PMC4959841

[B26] Kasthuri N. Hayworth K. J. Berger D. R. Schalek R. L. Conchello J. A. Knowles-Barley S. (2015). Saturated reconstruction of a volume of neocortex. Cell 162, 648–661. 10.1016/j.cell.2015.06.054 26232230

[B27] Katidou M. Vidaki M. Strigini M. Karagogeos D. (2008). The immunoglobulin superfamily of neuronal cell adhesion molecules: lessons from animal models and correlation with human disease. Biotechnol. J. 3, 1564–1580. 10.1002/biot.200800281 19072911

[B28] Kay J. N. Roeser T. Mumm J. S. Godinho L. Mrejeru A. Wong R. O. L. (2004). Transient requirement for ganglion cells during assembly of retinal synaptic layers. Development 131, 1331–1342. 10.1242/dev.01040 14973290

[B29] Kim I.-J. Zhang Y. Meister M. Sanes J. R. (2010). Laminar restriction of retinal ganglion cell dendrites and axons: subtype-specific developmental patterns revealed with transgenic markers. J. Neurosci. 30, 1452–1462. 10.1523/JNEUROSCI.4779-09.2010 20107072 PMC2822471

[B30] Kim J. Zhao T. Petralia R. S. Yu Y. Peng H. Myers E. (2011). mGRASP enables mapping mammalian synaptic connectivity with light microscopy. Nat. Methods 9, 96–102. 10.1038/nmeth.1784 22138823 PMC3424517

[B31] Krishnaswamy A. (2016). Building connections. Science 354:558. 10.1126/science.aak9763 27811260

[B32] Krishnaswamy A. Yamagata M. Duan X. Hong Y. K. Sanes J. R. (2015). Sidekick 2 directs formation of a retinal circuit that detects differential motion. Nature 524, 466–470. 10.1038/nature14682 26287463 PMC4552609

[B33] Langley J. N. (1892). On the origin from the spinal cord of the cervical and upper thoracic sympathetic fibres, with some observations on white and grey rami communications. Philos. Trans. R. Soc. London B 183, 85–124. 10.1098/rstb.1892.0002

[B34] Lefebvre J. L. Kostadinov D. Chen W. V. Maniatis T. Sanes J. R. (2012). Protocadherins mediate dendritic self-avoidance in the mammalian nervous system. Nature 488, 517–521. 10.1038/nature11305 22842903 PMC3427422

[B35] Lefebvre J. L. Sanes J. R. Kay J. N. (2015). Development of dendritic form and function. Annu. Rev. Cell Dev. Biol. 31, 741–777. 10.1146/annurev-cellbio-100913-013020 26422333

[B36] Luo L. Callaway E. M. Svoboda K. (2018). Genetic dissection of neural circuits: a decade of progress. Neuron 98, 256–281. 10.1016/j.neuron.2018.03.040 29673479 PMC5912347

[B37] Macosko E. Z. Basu A. Satija R. Nemesh J. Shekhar K. Goldman M. (2015). Highly parallel genome-wide expression profiling of individual ccells using nanoliter droplets. Cell 161, 1202–1214. 10.1016/j.cell.2015.05.002 26000488 PMC4481139

[B38] Marshel J. H. Kim Y. S. Machado T. A. Quirin S. Benson B. Kadmon J. (2019). Cortical layer-specific critical dynamics triggering perception. Science 365:eaaw5202. 10.1126/science.aaw5202 31320556 PMC6711485

[B39] Masland R. H. (2001). The fundamental plan of the retina. Nat. Neurosci. 4, 877–886. 10.1038/nn0901-877 11528418

[B40] Masland R. H. (2012). The neuronal organization of the retina. Neuron 76, 266–280. 10.1016/j.neuron.2012.10.002 23083731 PMC3714606

[B41] Matsuoka R. L. Chivatakarn O. Badea T. C. Samuels I. S. Cahill H. Katayama K.-I. (2011a). Class 5 transmembrane semaphorins control selective mammalian retinal lamination and function. Neuron 71, 460–473. 10.1016/j.neuron.2011.06.009 21835343 PMC3164552

[B42] Matsuoka R. L. Nguyen-Ba-Charvet K. T. Parray A. Badea T. C. Chedotal A. Kolodkin A. L. (2011b). Transmembrane semaphorin signalling controls laminar stratification in the mammalian retina. Nature 470, 259–263. 10.1038/nature09675 21270798 PMC3063100

[B43] Matsuoka R. L. Sun L. O. Katayama K. Yoshida Y. Kolodkin A. L. (2013). Sema6B, sema6c and sema6d expression and function during mammalian retinal development. PLoS One 8:e63207. 10.1371/journal.pone.0063207 23646199 PMC3640007

[B44] Menon K. P. Kulkarni V. Takemura S.-Y. Anaya M. Zinn K. (2019). Interactions between dpr11 and DIP-γ control selection of amacrine neurons in drosophila color vision circuits. Elife 8:e48935. 10.7554/elife.48935 31692445 PMC6879306

[B45] Miskevich F. Zhu Y. Ranscht B. Sanes J. R. (1998). Expression of multiple cadherins and catenins in the chick optic tectum. Mol. Cell. Neurosci. 12, 240–255. 10.1006/mcne.1998.0718 9828089

[B46] Morsut L. Roybal K. T. Xiong X. Gordley R. M. Coyle S. M. Thomson M. (2016). Engineering customized cell sensing and response behaviors using synthetic notch receptors. Cell 164, 780–791. 10.1016/j.cell.2016.01.012 26830878 PMC4752866

[B47] Nguyen D. N. Liu Y. Litsky M. L. Reinke R. (1997). The sidekick gene, a member of the immunoglobulin superfamily, is required for pattern formation in the drosophila eye. Development 124, 3303–3312. 9310325 10.1242/dev.124.17.3303

[B48] Packer A. M. Russell L. E. Dalgleish H. W. P. Häusser M. (2015). Simultaneous all-optical manipulation and recording of neural circuit activity with cellular resolution *in vivo*. Nat. Methods 12, 140–146. 10.1038/nmeth.3217 25532138 PMC4933203

[B49] Peng Y. R. Shekhar K. Yan W. Herrmann D. Sappington A. Bryman G. S. (2019). Molecular classification and comparative taxonomics of foveal and peripheral cells in primate retina. Cell 176, 1222.e22–1237.e22. 10.1016/j.cell.2019.01.004 30712875 PMC6424338

[B50] Peng Y. R. Tran N. M. Krishnaswamy A. Kostadinov D. Martersteck E. M. Sanes J. R. (2017). Satb1 regulates contactin 5 to pattern dendrites of a mammalian retinal ganglion cell. Neuron 95, 869.e6–883.e6. 10.1016/j.neuron.2017.07.019 28781169 PMC5575751

[B760] Randlett O. MacDonald R. B. Yoshimatsu T. Almeida A. D. Suzuki S. C. Wong R. O. (2013). Cellular Requirements for Building a Retinal Neuropil. Cell Rep. 3, 282–290. 10.1016/j.celrep.2013.01.020 23416047 PMC3607253

[B51] Ray T. A. Roy S. Kozlowski C. Wang J. Cafaro J. Hulbert S. W. (2018). Formation of retinal direction-selective circuitry initiated by starburst amacrine cell homotypic contact. Elife 7:e34241. 10.7554/eLife.34241 29611808 PMC5931800

[B52] Rheaume B. A. Jereen A. Bolisetty M. Sajid M. S. Yang Y. Renna K. (2018). Author correction: single cell transcriptome profiling of retinal ganglion cells identifies cellular subtypes. Nat. Commun. 9:3203. 10.1038/s41467-018-05792-3 30087343 PMC6081442

[B53] Sanes J. R. Masland R. H. (2015). The types of retinal ganglion cells: current status and implications for neuronal classification. Annu. Rev. Neurosci. 38, 221–246. 10.1146/annurev-neuro-071714-034120 25897874

[B54] Sanes J. R. Yamagata M. (2009). Many paths to synaptic specificity. Annu. Rev. Cell Dev. Biol. 25, 161–195. 10.1146/annurev.cellbio.24.110707.175402 19575668

[B55] Sanes J. R. Zipursky S. L. (2010). Design principles of insect and vertebrate visual systems. Neuron 66, 15–36. 10.1016/j.neuron.2010.01.018 20399726 PMC2871012

[B56] Schubert R. Trenholm S. Balint K. Kosche G. Cowan C. S. Mohr M. A. (2018). Virus stamping for targeted single-cell infection *in vitro* and *in vivo*. Nat. Biotechnol. 36, 81–88. 10.1038/nbt.4034 29251729

[B57] Shapiro L. Love J. Colman D. R. (2007). Adhesion molecules in the nervous system: structural insights into function and diversity. Annu. Rev. Neurosci. 30, 451–474. 10.1146/annurev.neuro.29.051605.113034 17600523

[B58] Shekhar K. Lapan S. W. Whitney I. E. Tran N. M. Macosko E. Z. Kowalczyk M. (2016). Comprehensive classification of retinal bipolar neurons by single-cell transcriptomics. Cell 166, 1308.e30–1323.e30. 10.1016/j.cell.2016.07.054 27565351 PMC5003425

[B59] Spampinato G. L. B. Ronzitti E. Zampini V. Ferrari U. Trapani F. Khabou H. (2019). All-optical interrogation of a direction selective retinal circuit by holographic wave front shaping. Biorxiv [preprint]. 10.1101/513192

[B60] Sperry R. W. (1943). Visuomotor coordination in the newt (triturus viridescens) after regeneration of the optic nerve. J. Comp. Neurol. 79, 33–55. 10.1002/cne.900790104

[B61] Stacy R. C. Wong R. O. L. (2003). Developmental relationship between cholinergic amacrine cell processes and ganglion cell dendrites of the mouse retina. J. Comp. Neurol. 456, 154–166. 10.1002/cne.10509 12509872

[B62] Sun L. O. Jiang Z. Rivlin-Etzion M. Hand R. Brady C. M. Matsuoka R. L. (2013). On and off retinal circuit assembly by divergent molecular mechanisms. Science 342:1241974. 10.1126/science.1241974 24179230 PMC3863450

[B63] Suzuki S. Sano K. Tanihara H. (1991). Diversity of the cadherin family: evidence for eight new cadherins in nervous tissue. Cell Regul. 2, 261–270. 10.1091/mbc.2.4.261 2059658 PMC361775

[B64] Suzuki S. C. Inoue T. Kimura Y. Tanaka T. Takeichi M. (1997). Neuronal circuits are subdivided by differential expression of type-II classic cadherins in postnatal mouse brains. Mol. Cell. Neurosci. 9, 433–447. 10.1006/mcne.1997.0626 9361280

[B65] Takeichi M. (1988). The cadherins: cell-cell adhesion molecules controlling animal morphogenesis. Development 102, 639–655. 3048970 10.1242/dev.102.4.639

[B66] Talay M. Richman E. B. Snell N. J. Hartmann G. G. Fisher J. D. Sorkaç A. (2017). Transsynaptic mapping of second-order taste neurons in flies by trans-tango. Neuron 96, 783.e4–795.e4. 10.1016/j.neuron.2017.10.011 29107518 PMC5693608

[B67] Tan L. Zhang K. X. Pecot M. Y. Nagarkar-Jaiswal S. Lee P.-T. Takemura S.-Y. (2015). Ig superfamily ligand and receptor pairs expressed in synaptic partners in drosophila. Cell 163, 1756–1769. 10.1016/j.cell.2015.11.021 26687360 PMC4804707

[B68] Tran N. M. Shekhar K. Whitney I. E. Jacobi A. Benhar I. Hong G. (2019). Single-cell profiles of retinal ganglion cells differing in resilience to injury reveal neuroprotective genes. Neuron 104, 1039.e12–1055.e12. 10.1016/j.neuron.2019.11.006 31784286 PMC6923571

[B69] Voinescu P. E. Kay J. N. Sanes J. R. (2009). Birthdays of retinal amacrine cell subtypes are systematically related to their molecular identity and soma position. J. Comp. Neurol. 517, 737–750. 10.1002/cne.22200 19827163 PMC2814066

[B70] Wässle H. Puller C. Müller F. Haverkamp S. (2009). Cone contacts, mosaics and territories of bipolar cells in the mouse retina. J. Neurosci. 29, 106–117. 10.1523/JNEUROSCI.4442-08.2009 19129389 PMC6664901

[B71] Wei W. Hamby A. M. Zhou K. Feller M. B. (2011). Development of asymmetric inhibition underlying direction selectivity in the retina. Nature 469, 402–406. 10.1038/nature09600 21131947 PMC3974627

[B72] Wickersham I. R. Feinberg E. H. (2012). New technologies for imaging synaptic partners. Curr. Opin. Neurobiol. 22, 121–127. 10.1016/j.conb.2011.12.001 22221865

[B73] Wickersham I. R. Finke S. Conzelmann K.-K. Callaway E. M. (2007a). Retrograde neuronal tracing with a deletion-mutant rabies virus. Nat. Methods 4, 47–49. 10.1038/nmeth999 17179932 PMC2755236

[B74] Wickersham I. R. Lyon D. C. Barnard R. J. O. Mori T. Finke S. Conzelmann K.-K. (2007b). Monosynaptic restriction of transsynaptic tracing from single, genetically targeted neurons. Neuron 53, 639–647. 10.1016/j.neuron.2007.01.033 17329205 PMC2629495

[B75] Williams M. E. Wilke S. A. Daggett A. Davis E. Otto S. Ravi D. (2011). Cadherin-9 regulates synapse-specific differentiation in the developing hippocampus. Neuron 71, 640–655. 10.1016/j.neuron.2011.06.019 21867881 PMC3272880

[B76] Xu C. Theisen E. Maloney R. Peng J. Santiago I. Yapp C. (2019). Control of synaptic specificity by establishing a relative preference for synaptic partners. Neuron 103, 865.e7–877.e7. 10.1016/j.neuron.2019.06.006 31300277 PMC6728174

[B77] Xu S. Xiao Q. Cosmanescu F. Sergeeva A. P. Yoo J. Lin Y. (2018). Interactions between the Ig-superfamily proteins DIP-α and dpr6/10 regulate assembly of neural circuits. Neuron 100, 1369.e6–1384.e6. 10.1016/j.neuron.2018.11.001 30467079 PMC7501880

[B78] Yamagata M. Sanes J. R. (2008). Dscam and sidekick proteins direct lamina-specific synaptic connections in vertebrate retina. Nature 451, 465–469. 10.1038/nature06469 18216854

[B79] Yamagata M. Sanes J. R. (2010). Synaptic localization and function of sidekick recognition molecules require MAGI scaffolding proteins. J. Neurosci. 30, 3579–3588. 10.1523/jneurosci.6319-09.2010 20219992 PMC2863080

[B80] Yamagata M. Sanes J. R. (2012). Transgenic strategy for identifying synaptic connections in mice by fluorescence complementation (GRASP). Front. Mol. Neurosci. 5:18. 10.3389/fnmol.2012.00018 22355283 PMC3280602

[B81] Yamagata M. Sanes J. R. (2018). Expression and roles of the immunoglobulin superfamily recognition molecule sidekick1 in mouse retina. Front. Mol. Neurosci. 11:485. 10.3389/fnmol.2018.00485 30687002 PMC6333872

[B82] Yamagata M. Weiner J. A. Sanes J. R. (2002). Sidekicks: synaptic adhesion molecules that promote lamina-specific connectivity in the retina. Cell 110, 649–660. 10.1016/s0092-8674(02)00910-8 12230981

[B83] Yonehara K. Balint K. Noda M. Nagel G. Bamberg E. Roska B. (2011). Spatially asymmetric reorganization of inhibition establishes a motion-sensitive circuit. Nature 469, 407–410. 10.1038/nature09711 21170022

[B84] Yonehara K. Farrow K. Ghanem A. Hillier D. Balint K. Teixeira M. (2013). The first stage of cardinal direction selectivity is localized to the dendrites of retinal ganglion cells. Neuron 79, 1078–1085. 10.1016/j.neuron.2013.08.005 23973208

[B85] Young R. W. (1985). Cell differentiation in the retina of the mouse. Anat. Rec. 212, 199–205. 10.1002/ar.1092120215 3842042

[B86] Zhang C. Kolodkin A. L. Wong R. O. James R. E. (2017). Establishing wiring specificity in visual system circuits: from the retina to the brain. Annu. Rev. Neurosci. 40, 395–424. 10.1146/annurev-neuro-072116-031607 28460185 PMC13321308

[B87] Zipursky S. L. Sanes J. R. (2010). Chemoaffinity revisited: dscams, protocadherins and neural circuit assembly. Cell 143, 343–353. 10.1016/j.cell.2010.10.009 21029858

